# Identification of genomic functional hotspots with copy number alteration in liver cancer

**DOI:** 10.1186/1687-4153-2013-14

**Published:** 2013-10-25

**Authors:** Tzu-Hung Hsiao, Hung-I Harry Chen, Stephanie Roessler, Xin Wei Wang, Yidong Chen

**Affiliations:** 1 Greehey Children's Cancer Research Institute, University of Texas Health Science Center at San Antonio, San Antonio, TX 78229, USA; 2 Institute of Pathology, University Hospital, Im Neuenheimer Feld 224, Room 2.034, Heidelberg 69120, Germany; 3 Laboratory of Human Carcinogenesis, National Cancer Institute, NIH, Bethesda, MD 20892, USA; 4 Department of Epidemiology and Biostatistics, University of Texas Health Science Center at San Antonio, San Antonio, TX 78229, USA

**Keywords:** Copy number alteration, Gene set enrichment, Pathway analysis, Liver cancer

## Abstract

Copy number alterations (CNAs) can be observed in most of cancer patients. Several oncogenes and tumor suppressor genes with CNAs have been identified in different kinds of tumor. However, the systematic survey of CNA-affected functions is still lack. By employing systems biology approaches, instead of examining individual genes, we directly identified the functional hotspots on human genome. A total of 838 hotspots on human genome with 540 enriched Gene Ontology functions were identified. Seventy-six aCGH array data of hepatocellular carcinoma (HCC) tumors were employed in this study. A total of 150 regions which putatively affected by CNAs and the encoded functions were identified. Our results indicate that two immune related hotspots had copy number alterations in most of patients. In addition, our data implied that these immune-related regions might be involved in HCC oncogenesis. Also, we identified 39 hotspots of which copy number status were associated with patient survival. Our data implied that copy number alterations of the regions may contribute in the dysregulation of the encoded functions. These results further demonstrated that our method enables researchers to survey biological functions of CNAs and to construct regulation hypothesis at pathway and functional levels.

## Introduction

Chromosomal instability is one of the characteristics in cancer [1] and results in the numerical and structural alterations of DNA copy number variations (CNAs). Recently, some literatures have reported the association of CNAs and patient survival in different tumors [2-4]. Several important oncogenes or tumor suppressors were also showed with high frequency of gain or loss status in different cancers. For example, the copy number amplification of gene Her2, which is the addicted oncogene in the HER2+ subtype of breast cancer, was highly correlated with the gene overexpression [5]. However, in addition to focal amplification, most tumors display multiple and broad ranges of copy number change, where large number of genes are involved in and potentially to be induced or suppressed due to copy number amplifications or deletions. Some *in vitro* studies were performed to survey the affected functions of CNAs [6-8]. For example, Nicole et al. utilized the shRNA library to identify the GO and STOP genes which positively and negatively regulate proliferation to evaluate the effect of gene deletions [7], respectively. They also proposed a model called 'Cancer Gene Island’, which encompasses high density of genes with the same function within a genomic region [7]. However, the *in vitro* studies were labor intensive if not cost prohibitive. Moreover, it is hard to perform a systematic analysis based on these approaches, thus, leaving the gene island model and their functions unexplored.

In conventional gene expression data analysis, several bioinformatics methods based on the concept of 'gene set enrichment analysis’ (GSEA) have been successfully utilized to explore the underlying molecular pathways and Gene Ontology functions [9-12]. The GSEA method assesses the number of overlap genes between two gene sets: the differentially expressed genes of a certain functional annotation and genes from the entire genome with the same annotation, to estimate the probability of the overlapping through the statistical test. The procedure provides a high throughput and systematic analysis to explore the putative activated pathways or functions.

Hepatocellular carcinoma (HCC) is one of the malignant cancers and the third leading cause of cancer death worldwide [13]. Major etiologies associated with HCC are hepatitis B virus (HBV) and hepatitis C virus (HCV) infection [14]. Previous studies have been reported in which comparative genomic hybridization by microarray (aCGH) was utilized to examine CNAs in HCC. Several regions with frequent copy number gain and loss were identified. The CNA-associated oncogenes and tumor suppressors, such as *MYC*, *JAG1*, *TP53*, and *RB1*, were also found [15-18]. The association between survival and CNAs has been investigated, and ten associated genes were reported [19]. However, the biological functions altered by CNAs remain unknown and thus need to be dissected.

According to the concept of Cancer Gene Island, here, we propose an algorithm to identify the spatial functional hotspots (SFHs) in human genome based on the enrichment analysis. The human genome is divided firstly into segments along the genomic sequence coordinate. Then, the tests of enrichment between the segments and whole genome functional categories are performed. Finally, a method which identifies the optimal regions of enriched functions between the segments was applied to examine putative SFHs. To demonstrate the ability of our method, we applied the method to an aCGH data set of HCC. The result showed several immune-related SFHs which showed gain and loss in HCC samples. Also, survival-associated SFHs were identified. The result also indicated that our system could serve as a useful method to understand the CNAs-affected functions.

## Methods

**Figure 1:**
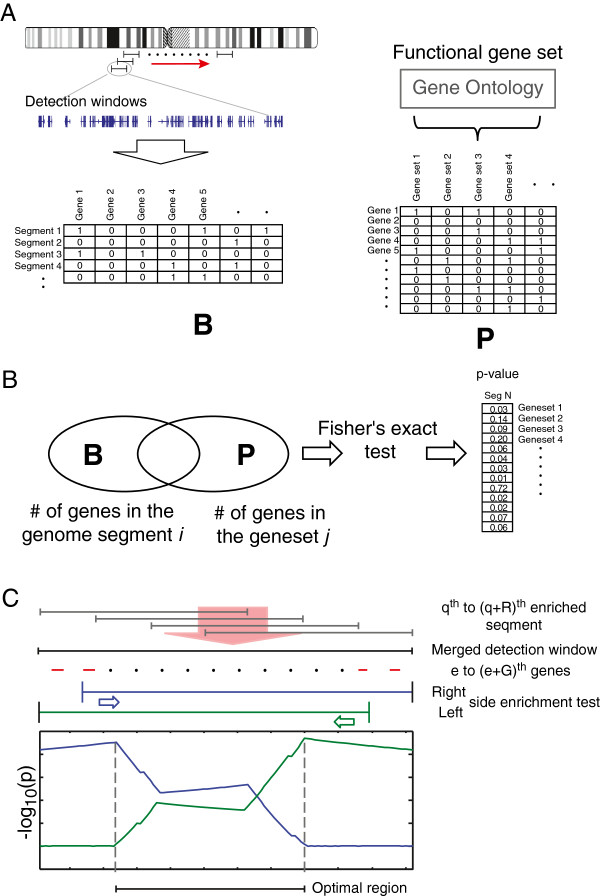
**Schematic diagram illustrating the enrichment analysis of spatial functional hotspots in human genome. (A)** The indicator matrix **B** was generated by sliding the detection window along the genome. It contains the information of genes located in each segment. The matrix **P** records the gene sets of Gene Ontology. **(B)** By comparing the two matrices assessed with Fisher's exact test, the *p* values of gene sets in each segment were generated. The enriched functions of each segment were then identified if passed the selection criteria. **(C)** The nearby segments with the same enriched function were merged to a detection window. The enrichment analysis between the function and the subset of genes in the windows were performed. The subset was constructed by excluding gene by gene along the left side or right side of the genome coordinate. The position with the smallest *p* value of left side and right side excluding subset was defined as the boundaries of the optimal region of the functional hotspot.

To identify the SFHs in human genome, we proposed a novel enrichment analysis that compares the genes contained within a genomic segment with all genes belonging to the same function categories associated to the genes within the segment under consideration based on the concept of gene set enrichment. As shown in Figure 1A, two matrixes, **B** and **P**, were constructed first. The indicator matrix **B** contains information whether or not a gene belongs to a genomic region (spatial segment) determined by a sliding window along the genomic position of all chromosomes or **B** = (*b*_*k,i*_) _*K*x*M*_, where *M* is the number of genes and *K* is the number of genome segments pre-determined and where *b*_*k,i*_ = 1 when *i*th gene is in the *k*th segment, otherwise 0. The matrix **P** = {*p*_*i,l*_}_*M*x*L*_ is also an indicator matrix of functional gene sets, where *L* is the number of functional gene sets and *p*_*i,l*_ = 1 when *i*th gene is in the *l*th GO (Gene Ontology) function, otherwise 0. The enrichment is defined as scoring function *C* of the two matrixes **B** and **P**.

(1) $$\text{ES}=C\left(B,P\right)$$

Here, we use Fisher's exact test as the score function *C*(**B**, **P**) (Figure 1B). Let $\;x_{k}=\overset{M}{\underset{i=1}{\sum }}b_{k,i}$ is the number of genes in the *k*th segment, $y_{l}=\overset{M}{\underset{i=1}{\sum }}p_{i,l}$ is the number of total genes in gene set *l*, and $\;z_{k,l}=\overset{M}{\underset{i=1}{\sum }}b_{k,i}p_{i,l}$ is the number of overlapped genes between the *k*th segment and gene set *l*. The *p* value of Fisher's exact test between the genome segment and the gene set can be calculated by

(2) $$P\left(x>z_{k,l}\right)=\overset{\infty }{\underset{h=z_{k,l}}{\sum }}\frac{\left(\begin{array}{c} x_{k}\\ h \end{array}\right)\left(\begin{array}{c} M-x_{k}\\ y_{l}-h \end{array}\right)}{\left(\begin{array}{c} M\\ y_{l} \end{array}\right)}$$

Based on the *p* values, we can determine if the function *l* was enriched at the genome segment *k*. Then, we merge and extend the enriched segments to a merged window to include all genes involved in the function *l* if the segments were located nearby and have position overlapping. As shown in Figure 1C, assuming *q*th to (*q* + *R*)th segments have enrichment for the function *l*, the genes involved in the merged windows can be expressed as vector ***d***:

(3) $$d_{i}=\left\{\begin{array}{cc} 1, & \mathrm{if}\;s_{i}>1\\ 0, & \;\mathrm{else} \end{array}\right)\;,$$

where $s_{i}=\overset{\left(q+R\right)}{\underset{t=q}{\sum }}b_{t,i}$. Assuming there are *G* genes (from *e*th to (*e* + *G*)th) located in the *q*th to (*q* + *R*)th enriched window, we defined the subsets of the *G* genes which exclude out genes gradually from left or right side according to the genome coordinate. Two parameters, *pL* and *pR*, which perform enrichment analysis (Fisher's exact test) between the subsets with the gene set of function *l* were introduced (Figure 1C). *pL* and *pR* are defined as:

(4) $$pR_{g}=C\left(\left\{d_{e+g},\ldots ,d_{\left(e+G\right)}\right\},p_{l}\right)\;$$

(5) $$pL_{g}=C\left(\left\{d_{e},\ldots ,d_{e+g}\right\},p_{l}\right),$$

where *g* = 1,…,*G* and *C*(.) is the enriched score function. Then, the optimal enriched region *o* of function *l* can be defined as:

(6) $$o=\left\{\text{argmin}\left(\text{pR}\right),\ldots ,\text{argmin}\left(\text{pL}\right)\right\}.$$

If the *p* value of the region *o* passed the selection threshold, *o* was defined as the SFHs of function *l*.

### Gene sets of genome segments and biological functions

To define the genome segments, the detection window size was set as one million base pairs (Mbp) after the testing of three different conditions (Additional file 1: Figure S1). The sliding distance was set at 0.25 Mbp. The genomic position of each gene was obtained from Ensembl (version Homo Sapiens 65) [20], or equivalent to NCBI human genome GRCh37. Therefore, a total of 12,098 segments were defined. To construct the functional gene sets, we downloaded all records of Gene Ontology from the BioMart website of Ensembl 65 (http://useast.ensembl.org/info/data/biomart.html) [20]. A total of 7,654 GO terms were downloaded. After excluding the gene sets containing fewer than 15 genes, 1,091, 404, and 275 gene sets associated to biological process (BP), molecular function (MF), and cellular component (CC) terms were utilized in this study, respectively.

### aCGH arrays of hepatocellular carcinoma

To identify the functional effect of CNAs in HCC, the aCGH array data set, GSE14322, was downloaded from GEO/NCBI website. The data set contains 76 HCC samples. The determination of CNAs was through the NEXUS software (BioDiscovery, San Diego, CA, USA). The CBS segmentation algorithm was performed to identify the segments of CNAs [21] using the thresholds of log2 values of fold change larger or smaller than ±0.2.

## Results

### Identification of spatial functional hotspots

**Figure 2:**
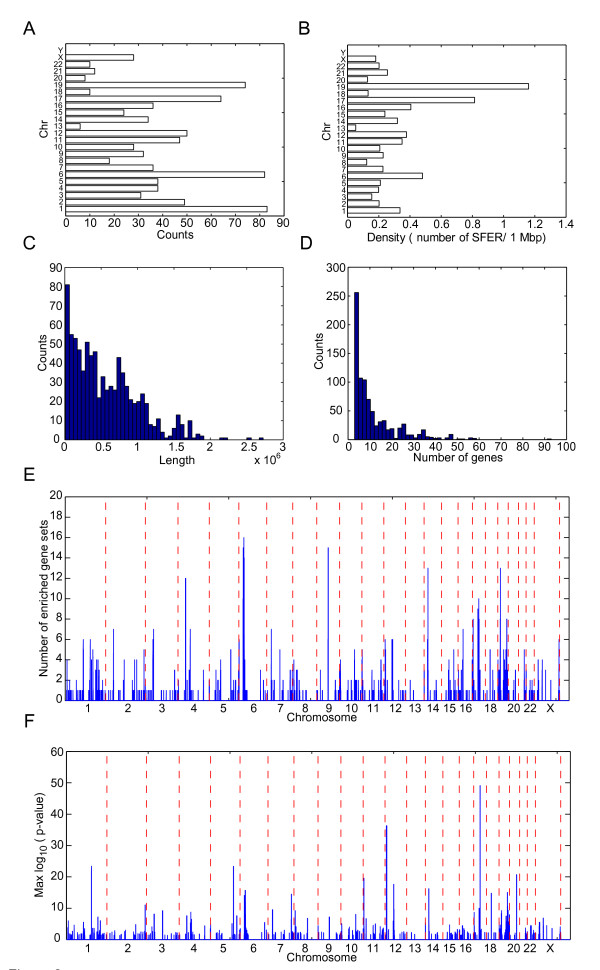
**The summary of the spatial functional hotspots. (A)** The histogram of the length of the SFHs. **(B)** The histogram of the gene numbers in the SFHs. **(C)** The SFH number in each chromosome. **(D)** The SFH density (numbers of SFHs per million base pair) in each chromosome. **(E)** The most significant *p* value and **(F)** the number of SFHs in the position of human genome. In **(E)** and **(F)**, the chromosomes were separated by red broken lines.

By using adjusted *p* values of Fisher's exact test < 0.05 after Bonferroni adjustment as the criteria, a total of 540 GO gene sets showed the functional enrichment in 838 SFHs. There are 443, 269, and 126 of SFHs belonging to BP, MF, and CC terms, respectively. On average, each chromosome contains 57 SFHs. Chromosome 1 has the largest number (147) of SFHs, and chromosome Y has no SFH (Figure 2A). The averaged SFH density is 0.43 SFHs per million base pairs (Mbp). Chromosome 6 has the highest SFH density (0.48 SFHs/Mbp) (Figure 2B). For the 838 SFHs, the average length of SFHs was 0.56 Mbp (Figure 2C) and the averaged 11.5 genes are in a SFH (Figure 2D). The SFH of 'sugar binding’ enrichment, which is located in the 7.88 to 10.6 Mbp region at chromosome 12, has the longest region length. The SFH of 'immune response’ enrichment (31.2 to 33 Mbp at chromosome 6), which contains 93 genes, has the largest number of genes. The region located in 29.7 to 31.5 Mbp at chromosome 6 contained the most number of enriched gene sets (16) (Figure 2E and Additional file 1: Table S1). The region includes lots of SFHs which have enrichment of immune-related gene sets, such like MHC class I protein complex, type I interferon-mediated signaling pathway, and immune response. Our finding indicated that the two regions are important for cell immunity.

**Figure 3:**
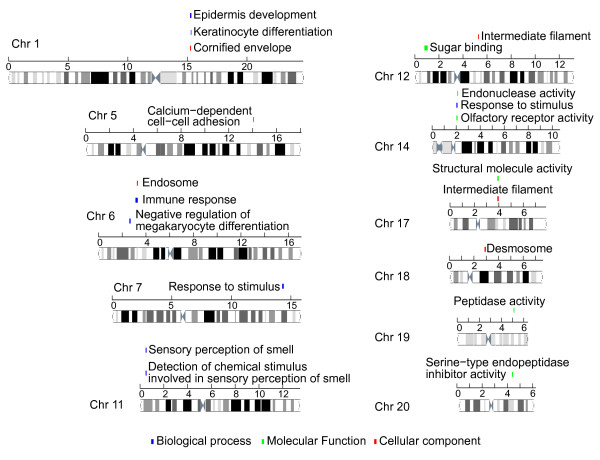
**The top 20 enriched function in human genome.** The top 20 significant SFHs, which contain 18 functions, were showed in the figure. The function of 'intermediate filament’ was enriched at both chromosomes 12 and 17. The 'response to stimulus’ function was enriched at chromosomes 7 and 14.

The smallest *p* values of the enriched gene sets in the genomic positions were showed in Figure 2F. There are nine SFHs which have adjusted *p* value smaller than 1 × 10^-20^. The 'intermediate filament’ gene set has the most significant adjusted *p* value (7.4 × 10^-50^) of the enrichment in the SFH of 38.8 to 39.4 Mbp region at chromosome 17. Twenty-nine out of 57 genes in the 'intermediate filament’ gene set are located at the hotspots. All of them belong to the keratin family genes, which are components of the cytoskeleton of epithelial cells. The top 20 SFHs were showed in Figure 3. Another SFH which is located at 52.6 to 53.3 Mbp region at chromosome 12 also showed the enrichment of intermediate filament. Other 12 keratin genes were contained. The SFH located at 31.2 to 33 Mbp regions of chromosome 6, which code 16 human leukocyte antigen (HLA) genes, enriched the immune response gene set. In summary, our result indicates that there are several functional hotspots within human genome related to the immune function.

### The affected function of copy number variation in liver cancer

To evaluate the effect function of CNAs in liver cancer, the dataset GSE14322, which contains 76 aCGH arrays of HCC samples, was downloaded and analyzed. The percentage of CNA status of each SFH was calculated. There are 61 and 89 SFHs that contained copy number gain and loss in more than 30% patients (25). The result was showed in Table S2 in Additional file 1, and the top ten SFHs were listed in Table 1. One immune-related gene set had the gain status in most of the samples (innate immune response), and one had loss status (response to virus), since the major etiologies of HCC are the infection of HBV and HCV. We hypothesize that those immune-related SFHs that harbor CNAs may play a role in the HCC carcinogenesis.

**Table 1:** Top ten gain/loss spatial functional hotspots (SFHs) of HCC with enriched functions

**Chr**	**Start pos**	**End pos**	**Number of CNVs**		**Presented gene set**	**Number gene sets**
Gain						
1	153.33	153.59	59	bp	Response to lipopolysaccharide	4
1	153.27	153.43	59	bp	*Innate immune response*	4
1	153.33	153.36	59	bp	Response to zinc ion	2
1	152.27	153.43	59	bp	Epidermis development	13
1	152.27	153.23	59	mf	Structural molecule activity	8
1	152.88	153.43	58	bp	Keratinocyte differentiation	12
1	152.88	153.23	58	bp	Peptide cross-linking	6
1	152.88	153.23	58	mf	Protein binding, bridging	4
1	153.50	153.60	57	mf	Protein homodimerization activity	5
1	153.50	153.60	57	cc	Perinuclear region of cytoplasm	5
Loss						
8	6.84	6.88	42	bp	*Response to virus*	3
4	190.39	191.01	40	mf	Sequence-specific DNA binding	6
4	190.39	191.01	40	mf	Sequence-specific DNA binding transcription Factor activity	6
8	6.35	6.91	40	cc	Extracellular space	7
4	90.80	91.76	40	cc	Platelet alpha granule lumen	2
4	90.80	91.76	40	bp	Platelet degranulation	2
8	26.61	27.47	39	bp	Response to stress	3
17	10.35	10.56	38	bp	Actin filament-based movement	2
8	26.37	27.31	37	bp	Response to cocaine	2
8	22.88	23.08	37	mf	Caspase activator activity	2

Immune-related functions are in italics.

**Figure 4:**
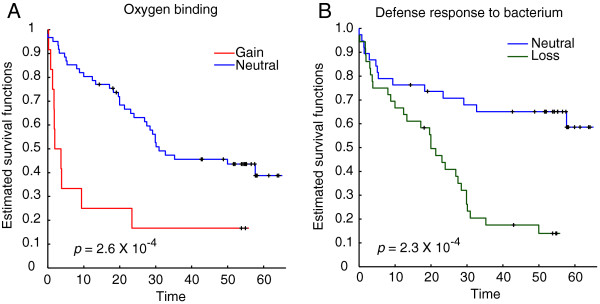
**Patient survival correlated to copy number status of tumor. (A)** Kaplan-Meier plots of patient disease-free survival were separated by the status of copy number in the SFH located at 41.1 to 41.9 Mbp at chromosome 19. The SFH has enrichment of 'oxygen binding’. Samples were assigned to two groups, copy number gain (red line) and neutral (blue line). The *p* values was statistically significant (<0.01). **(B)** Kaplan-Meier plots of the copy number status in SFH at 11.8 to 12.2 Mbp at chromosome 8. Samples were assigned to two groups, copy number loss (green line) and neutral (blue line). The SFH had enrichment of 'defense response to bacterium’. The *p* value of test was also significant.

We also analyzed the association between disease-free survival and the CNAs of the SFHs through log rank test. Using *p* < 0.01 as the threshold, a total of 20 and 19 SFHs of which gain and loss status were identified with survival association, respectively (see Table 2). The copy number gain status in the SFH which located at 41.1 to 41.9 Mbp at chromosome 19 had the smallest *p* value of the survival testing. The SFH had the enrichment of 'oxygen binding’. As shown in Figure 4A, the patients with copy number gain in the SFH had reduced survival comparing with neutral status. Interestingly, all the SFHs with survival-associated gain status were all located at chromosome 19 and ranged from 33.7 M to 59 Mbp. Four immune-related functions, defense response, regulation of immune response, antigen binding, and immune response, were enriched in the region. The finding indicated that the immune functional island located at the region is sensitive to patient survival. The SFH located at 11.8 to 12.2 Mbp at chromosome 8, which has enrichment of 'defense response to bacterium’, has the smallest *p* value of copy number loss status (Figure 4B). For SFHs with survival-associated loss status, 12 of them were located at 55 to 76.9 Mbp region at chromosome 4, and 7 of them were located at 11.1 to 38.3 Mbp at chromosome 8.

**Table 2:** The SFHs of which copy number status were associated with patient survival

**Chr**	**Start pos**	**End pos**	***p *** **value**		**Presented gene set**	**Number of overlapped genes**
Gain						
19	41.38	41.63	2.6E-04	mf	Oxygen binding	2
19	43.23	44.29	0.001	bp	Defense response	4
19	54.72	55.11	0.002	bp	Defense response	6
19	54.72	55.11	0.002	bp	Cell surface receptor linked Signaling pathway	6
19	40.09	40.23	0.003	mf	Lysophospholipase activity	2
19	40.09	40.23	0.003	mf	Carboxylesterase activity	2
19	39.41	39.52	0.004	cc	SCF ubiquitin ligase complex	2
19	39.41	39.52	0.004	mf	Glycoprotein binding	2
19	51.63	52.15	0.007	mf	Sugar binding	9
19	54.78	55.38	0.007	bp	Regulation of immune response	8
19	51.63	52.27	0.007	bp	Cell adhesion	11
19	42.18	44.32	0.007	cc	Anchored to membrane	9
19	54.72	55.55	0.007	mf	Transmembrane receptor activity	8
19	54.80	55.30	0.007	mf	Antigen binding	5
19	54.78	55.42	0.007	bp	Cellular defense response	5
19	54.72	55.55	0.007	cc	Integral to plasma membrane	12
19	58.55	59.08	0.007	bp	Viral reproduction	7
19	54.78	55.40	0.007	bp	Immune response	6
19	50.86	51.59	0.008	mf	Peptidase activity	17
19	45.41	45.45	0.008	mf	Lipid transporter activity	2
Loss						
8	11.83	12.18	2.4E-04	bp	Defense response to bacterium	4
4	55.10	55.99	0.001	bp	Vascular endothelial growth Factor receptor signaling pathway	2
4	55.10	55.99	0.001	mf	Growth factor binding	2
8	22.88	23.08	0.001	mf	Caspase activator activity	2
8	22.30	23.02	0.002	bp	Apoptosis	5
4	74.61	74.97	0.003	bp	Inflammatory response	5
8	26.37	27.47	0.004	cc	Growth cone	3
8	26.37	27.32	0.004	bp	Response to cocaine	2
8	22.01	23.08	0.005	bp	Cellular response to mechanical stimulus	3
4	68.69	69.36	0.006	mf	Serine-type endopeptidase activity	5
4	68.69	69.36	0.006	bp	Proteolysis	5
4	68.69	69.36	0.006	mf	Peptidase activity	5
4	70.86	71.40	0.007	bp	Biomineral tissue development	5
4	71.06	71.47	0.007	bp	Odontogenesis of dentine-containing tooth	3
4	74.26	74.85	0.007	cc	Platelet alpha granule lumen	3
4	74.26	74.85	0.007	bp	Platelet degranulation	3
4	76.92	76.94	0.008	bp	Defense response to virus	2
4	74.70	75.32	0.008	bp	Cell-cell signaling	4
8	38.13	38.33	0.008	bp	Cell growth	2

## Discussion

We introduced a system biology method, motivated by Cancer Gene Island, to identify the spatial functional hotspots in human genome. A statistical assay was presented to estimate the enrichment within genome regions to functional gene sets. By applying the terms of Gene Ontology into our method, the result provided the details of the function encoded in human genome. We set the two parameters of the algorithm, the length of window size and shift distance, as 1 and 0.25 Mbp, respectively. Although the setting of the parameters will affect the *p* value of enrichment testing for each segment, our algorithm performed an optimal procedure which merge the continual enriched segments and find the region with maximum *p* values by removing the gene one by one from both sides. Different settings of window size will not affect the results of final optimal regions. However, the detection of continual functional enriched segments could be missed under the condition of small window size because the windows contained no and less genes. To find out the workable parameters, we tested three conditions of window sizes, 0.5, 1, and 1.5 Mbp and found out that the condition of 0.5 Mbp contains large numbers of segments of which the gene number is less than three. The parameters of 1 and 1.5 Mbp contain fewer segments with low numbers of genes. Through the testing, we set the window size as 1 Mbp to analyze the human genome.

We applied the method in HCC data set to estimate the effect of hotspots in the genome. Using the data set GSE14322 as an example, a total of 150 SFHs have been identified with copy number alterations in most of the HCC patients, and the novelty of our analysis is to identify the functional hotspots in human genome. The region we identified is located with high density of genes that share the same biological function, and as we demonstrated in the HCC dataset, these functions may also be sensitive to CNAs. Two immune-related functional regions were identified with gain or loss in most of patients in the HCC dataset. The major carcinogenesis of HCC is the chronic and acute inflammation under HBV or HCV infection; thus, we hypothesize that these two regions we identified may also play a role in HCC oncogenesis.

We also identified 39 SFHs of which the copy number status was associated with patient survival. The result indicates that the copy number alterations in these regions may affect the function of tumor progression and then reflect on patient survival. For example, the patients who have copy number loss in the SFH which was enriched in inflammatory response have shorter survival. The chronic and acute inflammations induced by HBV and HCV infection have been proved to play an important role in HCC tumorgenesis [22,23]. Our data implied that copy number alterations may contribute in the inflammatory response. Also, other enriched functions in survival-related SFHs have been reported, such as regulation of immune response, cell growth, apoptosis, and caspase activator activity. The SFHs and enriched function we identified provided the clues of the association between CNAs and the regulations of the enriched functions. We expected that the SFHs we identified will provide further insight of affected functions of CNAs to uncover the mechanism of cancer.

## Conclusions

In this paper, we systematically surveyed human genome and identified 838 functional hotspots based on Gene Ontology classification. To substantiate our findings, 76 HCC tumors and their DNA copy number gain/loss statuses were examined closely. Among the 838 hotspots, a total of 150 regions affected by CNAs, and the encoded enriched functions were identified. Our results indicate that two immune-related hotspots had copy number alterations in most of the patients and might be involved in HCC oncogenesis. In addition, 39 survival-related hotspots were identified. Taken together, our results demonstrated that the method presented in the paper is a powerful tool to survey biological functions of CNAs and to construct regulation hypothesis at pathway and functional levels.

## Competing interests

All authors declare that they have no competing interests.

## Supplementary Material

Additional file 1 **Supplemental materials.** This file contains tables and a figure showing the enriched functions, spatial functional hotspots, and histogram of the gene numbers. Click here for file
