# Supplementary material for: Identification of genomic functional hotspots with copy number alteration in liver cancer
Source: EURASIP J Bioinform Syst Biol. 2013 Oct 25;2013(1):14. doi: 10.1186/1687-4153-2013-14 (PMC3833309; doi:10.1186/1687-4153-2013-14)
Supplement: Additional file 1 — Supplemental materials. This file contains tables and a figure showing the enriched functions, spatial functional hotspots, and histogram of the gene numbers. [file 1687-4153-2013-14-S1.docx]

**Supplemental Materials**

**Identification of Genomic Functional Hotspots**

**with Copy Number Alteration in Liver Cancer**

Tzu-Hung Hsiao, Hung-I Harry Chen, Stephanie Roessler, Xin Wei Wang, and Yidong Chen

Table S1: The region with 16 enriched functions.

| Region | Gene set |
| --- | --- |
| Chr 6 29.7-31.5Mbp | MHC class I protein complex |
|  | antigen processing and presentation |
|  | integral to lumenal side of endoplasmic reticulum membrane |
|  | MHC class I receptor activity |
|  | regulation of immune response |
|  | ER to Golgi transport vesicle membrane |
|  | integral to lumenal side of endoplasmic reticulum membrane |
|  | MHC class I receptor activity |
|  | MHC class I protein complex |
|  | ER to Golgi transport vesicle membrane |
|  | antigen processing and presentation |
|  | type I interferon-mediated signaling pathway |
|  | interferon-gamma-mediated signaling pathway |
|  | antigen processing and presentation of peptide antigen via MHC class I |
|  | regulation of immune response |
|  | intracellular |

Table S2: The spatial functional hotspots (SFHs) of HCC with copy number gain and the enriched functions.

| Chr | Start pos | End pos | # of CNVs | GO | Presented gene set | # gene sets |
| --- | --- | --- | --- | --- | --- | --- |
| 1 | 153.33 | 153.59 | 59 | bp | response to lipopolysaccharide | 4 |
| 1 | 153.27 | 153.43 | 59 | bp | innate immune response | 4 |
| 1 | 153.33 | 153.36 | 59 | bp | response to zinc ion | 2 |
| 1 | 152.27 | 153.43 | 59 | bp | epidermis development | 13 |
| 1 | 152.27 | 153.23 | 59 | mf | structural molecule activity | 8 |
| 1 | 152.88 | 153.43 | 58 | bp | keratinocyte differentiation | 12 |
| 1 | 152.88 | 153.23 | 58 | bp | peptide cross-linking | 6 |
| 1 | 152.88 | 153.23 | 58 | mf | protein binding, bridging | 4 |
| 1 | 153.51 | 153.60 | 57 | mf | protein homodimerization activity | 5 |
| 1 | 153.51 | 153.63 | 57 | cc | perinuclear region of cytoplasm | 5 |
| 1 | 153.51 | 153.52 | 57 | mf | calcium-dependent protein binding | 2 |
| 1 | 152.27 | 153.12 | 57 | cc | cornified envelope | 11 |
| 1 | 151.23 | 151.74 | 57 | bp | regulation of cellular amino acid metabolic process | 3 |
| 1 | 149.80 | 149.83 | 54 | bp | negative regulation of megakaryocyte differentiation | 2 |
| 1 | 149.78 | 149.90 | 54 | cc | nucleoplasm | 6 |
| 1 | 171.06 | 171.18 | 51 | bp | drug metabolic process | 2 |
| 1 | 160.51 | 161.19 | 51 | bp | leukocyte migration | 5 |
| 1 | 160.45 | 161.68 | 51 | mf | receptor activity | 14 |
| 1 | 168.51 | 169.70 | 51 | cc | extracellular space | 6 |
| 1 | 160.51 | 161.06 | 51 | bp | cell adhesion | 5 |
| 1 | 160.58 | 161.19 | 51 | cc | external side of plasma membrane | 4 |
| 1 | 169.07 | 169.70 | 49 | bp | leukocyte migration | 4 |
| 1 | 169.56 | 169.70 | 49 | bp | heterophilic cell-cell adhesion | 2 |
| 1 | 169.07 | 170.05 | 49 | bp | blood coagulation | 5 |
| 1 | 169.56 | 169.70 | 49 | bp | leukocyte cell-cell adhesion | 2 |
| 1 | 143.77 | 144.36 | 49 | mf | peptidyl-prolyl cis-trans isomerase activity | 2 |
| 1 | 196.62 | 196.80 | 48 | bp | complement activation | 2 |
| 1 | 203.15 | 203.48 | 47 | mf | extracellular matrix structural constituent | 3 |
| 1 | 192.13 | 193.22 | 46 | bp | positive regulation of GTPase activity | 5 |
| 1 | 192.13 | 193.22 | 46 | mf | GTPase activator activity | 5 |
| 1 | 192.13 | 193.22 | 46 | mf | signal transducer activity | 5 |
| 1 | 192.13 | 192.78 | 46 | bp | negative regulation of signal transduction | 3 |
| 1 | 186.41 | 186.96 | 46 | bp | decidualization | 2 |
| 1 | 186.41 | 186.96 | 46 | bp | response to vitamin D | 2 |
| 1 | 186.41 | 186.96 | 46 | bp | response to organic nitrogen | 2 |
| 1 | 206.64 | 207.97 | 46 | bp | innate immune response | 8 |
| 1 | 201.33 | 201.40 | 46 | bp | regulation of muscle contraction | 2 |
| 1 | 201.33 | 201.40 | 46 | bp | ventricular cardiac muscle tissue morphogenesis | 2 |
| 1 | 206.94 | 208.08 | 46 | cc | extracellular region | 10 |
| 1 | 207.08 | 208.42 | 46 | mf | receptor activity | 8 |
| 1 | 201.33 | 201.40 | 46 | bp | muscle filament sliding | 2 |
| 1 | 201.16 | 201.35 | 46 | cc | sarcomere | 2 |
| 1 | 221.87 | 222.89 | 46 | bp | negative regulation of JNK cascade | 2 |
| 1 | 220.14 | 220.32 | 46 | bp | tRNA aminoacylation for protein translation | 2 |
| 1 | 226.07 | 226.13 | 46 | bp | cell growth | 2 |
| 1 | 211.84 | 212.97 | 46 | cc | chromosome | 4 |
| 1 | 228.19 | 228.59 | 45 | bp | negative regulation of neurogenesis | 2 |
| 1 | 243.29 | 244.01 | 45 | cc | centriole | 2 |
| 1 | 228.60 | 228.68 | 45 | bp | protein autoubiquitination | 2 |
| 1 | 230.19 | 230.83 | 44 | cc | Golgi stack | 2 |
| 8 | 81.88 | 82.76 | 35 | mf | lipid binding | 6 |
| 8 | 79.43 | 79.72 | 33 | bp | negative regulation of catalytic activity | 2 |
| 6 | 31.87 | 32.00 | 28 | bp | complement activation | 4 |
| 6 | 31.78 | 32.10 | 28 | bp | response to unfolded protein | 4 |
| 6 | 31.87 | 32.00 | 28 | bp | complement activation, classical pathway | 3 |
| 6 | 31.95 | 32.00 | 28 | mf | endopeptidase inhibitor activity | 2 |
| 6 | 31.87 | 32.15 | 28 | bp | innate immune response | 5 |
| 8 | 61.59 | 62.63 | 27 | bp | palate development | 2 |
| 6 | 42.12 | 42.69 | 27 | bp | visual perception | 3 |
| 6 | 31.50 | 31.77 | 25 | mf | U6 snRNA binding | 2 |
| 6 | 36.00 | 37.00 | 25 | bp | nerve growth factor receptor signaling pathway | 4 |

Figure S3: The spatial functional hotspots (SFHs) of HCC with copy number loss and the enriched functions.

| Chr | Start pos | End pos | # of CNVs | GO | Presented gene set | # gene sets |
| --- | --- | --- | --- | --- | --- | --- |
| 23 | 23.67 | 26.19 | 67 | bp | spermatogenesis | 9 |
| 23 | 23.67 | 24.56 | 67 | bp | mRNA processing | 6 |
| 23 | 23.67 | 24.56 | 67 | bp | RNA splicing | 6 |
| 23 | 26.19 | 27.20 | 67 | bp | spermatogenesis | 4 |
| 23 | 25.13 | 25.44 | 67 | bp | single fertilization | 2 |
| 23 | 19.99 | 20.14 | 67 | mf | histone acetyltransferase activity | 2 |
| 23 | 26.76 | 27.20 | 67 | bp | single fertilization | 2 |
| 23 | 69.26 | 69.46 | 66 | bp | lipid biosynthetic process | 3 |
| 23 | 69.26 | 69.46 | 66 | mf | acyltransferase activity | 3 |
| 23 | 68.84 | 69.46 | 66 | cc | endoplasmic reticulum membrane | 4 |
| 23 | 22.74 | 24.56 | 66 | mf | RNA binding | 9 |
| 23 | 48.82 | 49.60 | 65 | cc | cellular_component | 10 |
| 23 | 48.83 | 49.60 | 65 | bp | biological_process | 10 |
| 23 | 48.83 | 49.60 | 65 | mf | molecular_function | 9 |
| 23 | 49.18 | 49.46 | 65 | bp | cellular defense response | 3 |
| 23 | 47.86 | 47.99 | 64 | bp | cell wall macromolecule catabolic process | 2 |
| 23 | 47.86 | 47.99 | 64 | mf | hydrolase activity, acting on glycosyl bonds | 2 |
| 23 | 47.00 | 48.44 | 63 | mf | nucleic acid binding | 15 |
| 23 | 101.09 | 102.35 | 61 | bp | mRNA export from nucleus | 4 |
| 23 | 100.66 | 101.73 | 61 | cc | actin cytoskeleton | 4 |
| 23 | 151.12 | 151.62 | 57 | bp | gamma-aminobutyric acid signaling pathway | 2 |
| 23 | 153.24 | 155.01 | 57 | mf | molecular_function | 10 |
| 23 | 150.91 | 151.83 | 56 | mf | ion channel activity | 4 |
| 23 | 148.66 | 149.93 | 56 | cc | cellular_component | 5 |
| 23 | 151.12 | 151.83 | 55 | mf | GABA-A receptor activity | 3 |
| 23 | 151.12 | 151.83 | 55 | mf | extracellular ligand-gated ion channel activity | 3 |
| 23 | 151.12 | 151.83 | 55 | cc | chloride channel complex | 3 |
| 23 | 151.12 | 151.83 | 55 | mf | chloride channel activity | 3 |
| 8 | 6.84 | 6.88 | 42 | bp | response to virus | 3 |
| 4 | 190.39 | 191.01 | 40 | mf | sequence-specific DNA binding | 6 |
| 4 | 190.39 | 191.01 | 40 | mf | sequence-specific DNA binding transcription factor activity | 6 |
| 8 | 6.36 | 6.91 | 40 | cc | extracellular space | 7 |
| 4 | 90.80 | 91.76 | 40 | cc | platelet alpha granule lumen | 2 |
| 8 | 26.61 | 27.47 | 39 | bp | response to stress | 3 |
| 17 | 10.35 | 10.56 | 38 | bp | actin filament-based movement | 2 |
| 8 | 26.37 | 27.32 | 37 | bp | response to cocaine | 2 |
| 8 | 22.88 | 23.08 | 37 | mf | caspase activator activity | 2 |
| 8 | 26.37 | 27.47 | 37 | cc | growth cone | 3 |
| 8 | 11.83 | 12.18 | 37 | bp | defense response to bacterium | 4 |
| 4 | 123.37 | 123.84 | 37 | mf | growth factor activity | 3 |
| 8 | 22.30 | 23.02 | 36 | bp | apoptosis | 5 |
| 17 | 3.00 | 3.30 | 36 | bp | detection of chemical stimulus involved in sensory perception of smell | 5 |
| 17 | 3.00 | 3.30 | 36 | bp | sensory perception of smell | 4 |
| 4 | 154.62 | 156.14 | 35 | bp | signal transduction | 6 |
| 8 | 6.78 | 7.27 | 35 | bp | defense response | 7 |
| 8 | 6.73 | 7.27 | 35 | bp | chemotaxis | 4 |
| 4 | 83.27 | 83.38 | 35 | cc | heterogeneous nuclear ribonucleoprotein complex | 2 |
| 8 | 22.01 | 23.08 | 34 | bp | cellular response to mechanical stimulus | 3 |
| 4 | 155.48 | 156.73 | 34 | bp | platelet activation | 5 |
| 4 | 155.48 | 156.73 | 34 | bp | blood coagulation | 5 |
| 17 | 8.34 | 9.15 | 34 | bp | neuron migration | 3 |
| 17 | 8.34 | 9.15 | 34 | bp | positive regulation of axon extension | 2 |
| 17 | 3.00 | 3.82 | 32 | mf | receptor activity | 12 |
| 17 | 3.47 | 3.87 | 32 | bp | cation transport | 4 |
| 17 | 3.41 | 3.82 | 32 | mf | cation channel activity | 3 |
| 17 | 3.41 | 3.82 | 32 | mf | calcium channel activity | 3 |
| 17 | 3.41 | 3.82 | 32 | mf | ion channel activity | 4 |
| 17 | 3.47 | 4.27 | 32 | mf | ATP binding | 8 |
| 17 | 3.47 | 3.87 | 32 | bp | transport | 5 |
| 16 | 50.35 | 50.84 | 31 | bp | Wnt receptor signaling pathway | 3 |
| 4 | 70.86 | 71.40 | 30 | bp | biomineral tissue development | 5 |
| 4 | 71.06 | 71.47 | 30 | bp | odontogenesis of dentine-containing tooth | 3 |
| 17 | 3.80 | 4.85 | 30 | bp | platelet activation | 5 |
| 17 | 0.01 | 0.26 | 30 | bp | exocytosis | 2 |
| 4 | 68.69 | 69.36 | 29 | mf | serine-type endopeptidase activity | 5 |
| 4 | 68.69 | 69.36 | 29 | bp | proteolysis | 5 |
| 4 | 68.69 | 69.36 | 29 | mf | peptidase activity | 5 |
| 16 | 67.92 | 68.03 | 29 | cc | anchored to membrane | 3 |
| 17 | 5.02 | 5.29 | 29 | cc | recycling endosome | 2 |
| 4 | 74.61 | 74.97 | 28 | bp | inflammatory response | 5 |
| 4 | 74.85 | 75.72 | 28 | bp | positive regulation of cell division | 3 |
| 4 | 74.86 | 75.72 | 28 | bp | positive regulation of cell proliferation | 4 |
| 4 | 76.92 | 76.94 | 28 | bp | defense response to virus | 2 |
| 16 | 56.90 | 57.02 | 28 | cc | vesicle | 2 |
| 4 | 74.26 | 74.85 | 27 | cc | platelet alpha granule lumen | 3 |
| 4 | 74.26 | 74.85 | 27 | bp | platelet degranulation | 3 |
| 4 | 74.70 | 75.32 | 27 | bp | cell-cell signaling | 4 |
| 4 | 74.74 | 75.72 | 27 | mf | growth factor activity | 6 |
| 16 | 86.54 | 86.60 | 27 | bp | mesoderm development | 2 |
| 16 | 86.54 | 86.60 | 27 | bp | somitogenesis | 2 |
| 16 | 86.54 | 86.60 | 27 | bp | vasculogenesis | 2 |
| 16 | 86.54 | 86.60 | 27 | bp | blood vessel development | 2 |
| 17 | 0.91 | 1.42 | 27 | bp | actin cytoskeleton organization | 3 |
| 17 | 0.91 | 1.36 | 27 | bp | nerve growth factor receptor signaling pathway | 3 |
| 16 | 68.11 | 68.87 | 26 | bp | cellular response to lithium ion | 2 |
| 8 | 38.13 | 38.33 | 25 | bp | cell growth | 2 |
| 8 | 38.76 | 38.96 | 25 | mf | laminin binding | 2 |
| 17 | 4.84 | 4.86 | 25 | bp | gluconeogenesis | 2 |
| 13 | 48.63 | 49.06 | 25 | bp | androgen receptor signaling pathway | 2 |


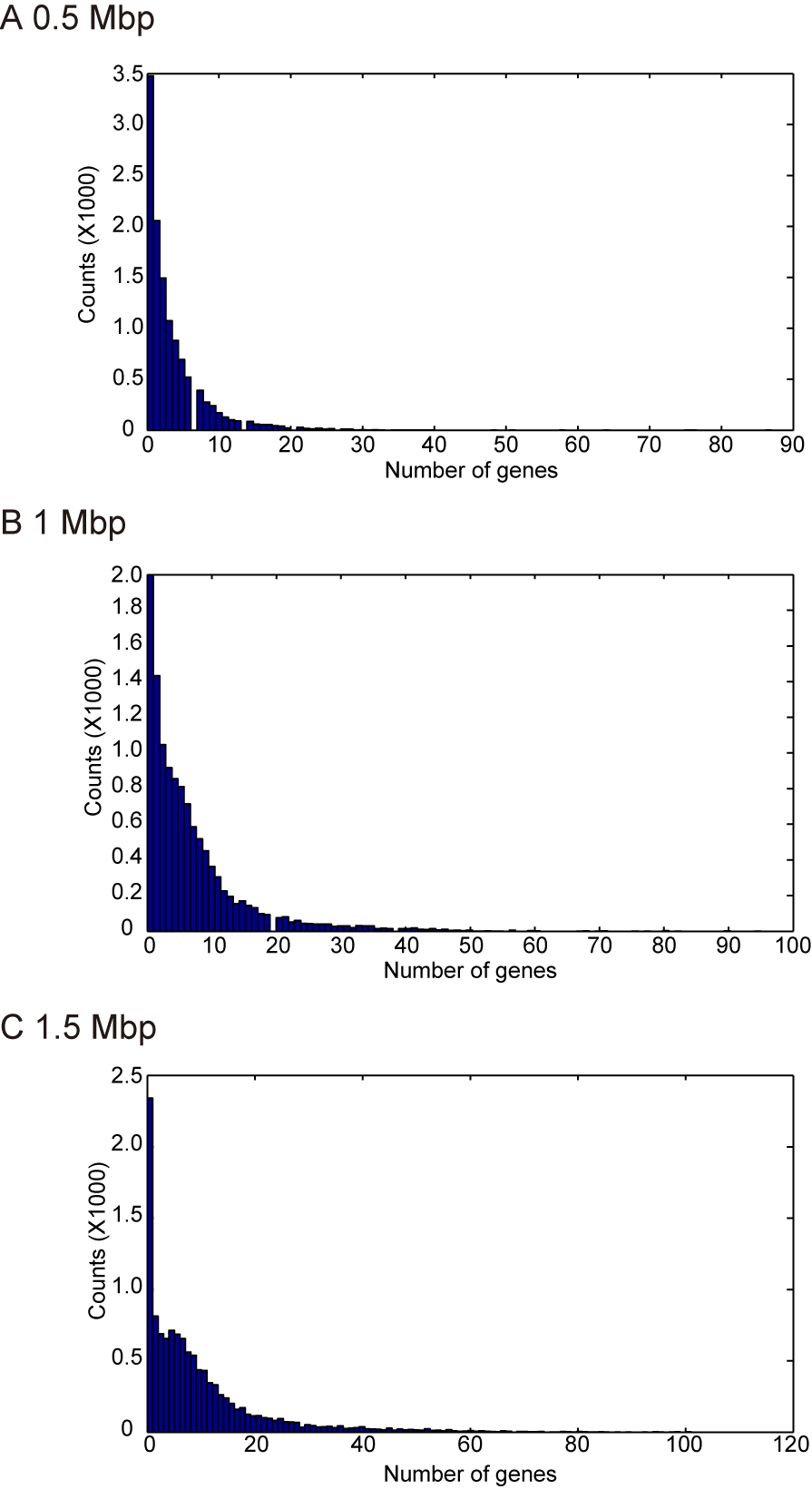


**Figure S1. Histogram of gene numbers in the detecting windows.** Three conditions of window size, 0.5Mbp, 1Mbp, and 1.5Mbp, were tested. The averaged numbers of gene in each window were 3.4, 6.8, and 10.1 for the conditions of 0.5Mbp, 1Mbp, and 1.5Mbp, respectively. A total of 7,030, 4,478, 3,155 windows contain less than 3 genes for 0.5Mbp, 1Mbp, and 1.5Mbp setting, respectively.
